# Overview of Novel Antipsychotic Drugs: State of the Art, New Mechanisms, and Clinical Aspects of Promising Compounds

**DOI:** 10.3390/biomedicines13010085

**Published:** 2025-01-01

**Authors:** Letizia Biso, Marco Carli, Marco Scarselli, Biancamaria Longoni

**Affiliations:** Department of Translational Research and New Technologies in Medicine and Surgery, University of Pisa, 56126 Pisa, Italy; l.biso@studenti.unipi.it (L.B.); carlimarco@outlook.it (M.C.); marco.scarselli@unipi.it (M.S.)

**Keywords:** novel antipsychotics, schizophrenia, clinical trials

## Abstract

Antipsychotic medications are a vast class of drugs used for the treatment of psychotic disorders such as schizophrenia. Although numerous compounds have been developed since their introduction in the 1950s, several patients do not adequately respond to current treatments, or they develop adverse reactions that cause treatment discontinuation. Moreover, in the past few decades, discoveries in the pathophysiology of psychotic disorders have opened the way for experimenting with novel compounds that have alternative mechanisms of action, with some of them showing promising results in early trials. The scope of this review was to summarize the novel antipsychotics developed, their current experimental status, and their mechanisms of action. In particular, we analyzed the main classes of investigational antipsychotics, such as monoamine, glutamate, acetylcholine, cannabinoid receptor modulators, enzyme inhibitors, ion channel modulators, and mixed receptor modulators. In addition, the safety profiles and adverse effects of these drugs were carefully evaluated, considering the relevance of these aspects for patients’ drug adherence and quality of life, especially in the long-term treatment. Lastly, we tried to understand which compounds have greater potential to be approved by the principal drug regulatory agencies in the next years and if they could be used for diseases other than psychotic disorders.

## 1. Introduction

Antipsychotic medications remain the main treatment for schizophrenia, both in an acute setting and in maintenance treatment [1,2]. Antipsychotics are also indicated for the treatment of mood disorders, and they are used for different conditions and in various populations (including the pediatric and the elderly populations), both in approved and off-label settings [3,4,5,6,7].

The first antipsychotic drug that was introduced in 1950 was chlorpromazine, a compound of the phenothiazine family [8]. Since then, many other drugs have been commercialized as antipsychotic medications, with more or less similar mechanisms of action. The main difference between first (“typical”) and second (“atypical”) generation antipsychotics is that while typical antipsychotics mainly act as D2 dopaminergic receptor blockers, atypical antipsychotics are characterized by the ability to inhibit both 5-HT2A serotoninergic and D2 dopaminergic receptors and, in general, are characterized by the involvement of different molecular targets [9,10,11,12,13]. In the last few decades, third-generation antipsychotics were introduced, and their primary mechanism of action is the partial D2 receptor agonism [14].

Despite the vast number of drugs that have been used since the 1950s, not all patients adequately respond to medications. Although difficult to estimate, it is thought that 20–50% of patients with schizophrenia do not respond to commonly used antipsychotics and are, therefore, considered treatment-resistant [15]. To date, clozapine is considered the most effective choice for treatment-resistant schizophrenia, but even in this case, 40–70% of patients do not benefit from clozapine treatment (clozapine-resistant schizophrenia) [16].

**Figure 1:**
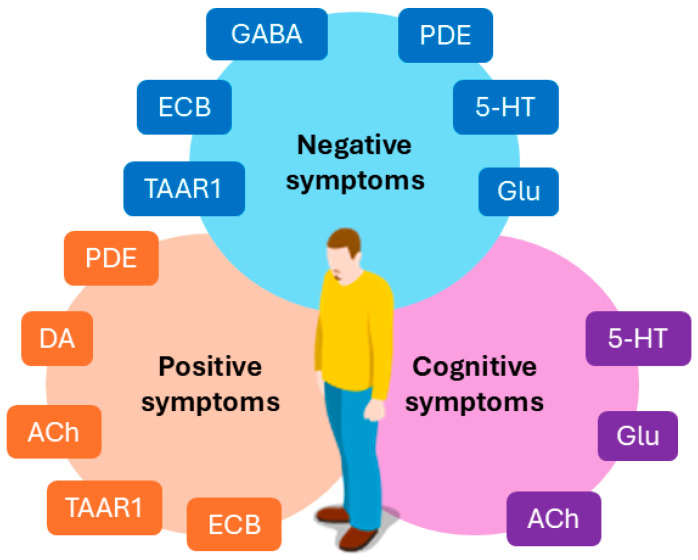
An overview of the neurotransmitters involved in positive, negative, and cognitive symptoms in schizophrenia. GABA: γ-aminobutyric acid; DA: dopamine; Glu: glutamate; ACh: acetylcholine; 5-HT: serotonin; TAAR1: trace amine-associated receptor 1; PDE: phosphodiesterase enzymes; ECB: endocannabinoid system.

However, in the case of treatment response, the quality of life of patients with schizophrenia remains lower than the healthy population, despite proper adherence to treatment and the addition of non-pharmacological interventions [17]. The most impairing symptoms, which are also the least responsive to treatment, are negative and cognitive symptoms that deeply impact the quality of life and contribute to the chronic disability that characterizes schizophrenia [18]. Negative symptoms represent a core component of schizophrenia and are characterized by a decrease in motivation, expression, and interests (avolition, anhedonia, blunted effects, alogia, socialization deficits) [19]. Cognitive symptoms are multifactorial, and their pathogenesis is linked to multiple neurotransmitters systems, impairing various domains of cognition, including memory, learning, and executive functions [20]. Moreover, both first- and second-generation antipsychotics are burdened by important adverse effects, that can impact the patients’ life [21,22]. Figure 1 summarizes the primary neurotransmitters involved in positive, negative, and cognitive symptoms in schizophrenia.

With these premises, it is not surprising that numerous trials involving new compounds with putative antipsychotic properties and novel mechanisms of action have recently been conducted. The scope of this review was to analyze the main novel compounds categories and explore the most promising drugs that have not been approved in the market yet but that may constitute the next generation of antipsychotics for the treatment of schizophrenia. These new drugs, besides reducing positive symptoms, target especially negative and cognitive symptoms of schizophrenia; therefore, they could constitute a valid instrument for the clinician, if they eventually reach the market, given the burden that these symptoms represent for patients. The main objective is to focus on compounds that reached phase III clinical trials, or earlier research stages, by searching both in published literature databases and main clinical trial registers. For drugs failing clinical trials on schizophrenia, but showing promising results for other neuropsychiatric disorders, we added a brief discussion on potential alternative applications.

## 2. Monoamine Receptor Modulators

### 2.1. TAAR1 Agonists

In the past decade, researchers tried to develop novel drugs with antipsychotic properties that could modulate monoamine levels beyond the traditional D2/5HT2 receptors blockade. One of the most important compounds is ulotaront (SEP-363856). Ulotaront is a trace amine-associated receptor 1 (TAAR1) agonist which also is an agonist on 5-HT1A receptors and modulates glutamatergic signaling [23]. Trace amines are endogenous messengers similar to the main amine neurotransmitters but found at very low concentrations in the central nervous system (CNS) [24]. TAAR1 is a G-protein-coupled receptor (GPCR) that was discovered in vertebrates in 2001, and since then, it has been proposed as a target for the treatment of several psychiatric conditions, given its ability to be activated by monoamine neurotransmitters (dopamine, serotonin, and norepinephrine) and their metabolites [25,26].

Ulotaront is a highly permeable, highly soluble, small molecule of the thienopyrans class. It was synthesized by condensating 2-(thiophen-3-yl)ethanol and *N*-methylaminoacetaldehyde dimethyl acetal with triflic acid presence in dimethoxyethane, and it has a molecular weight (MW) of 183.27 g/mol [27,28]. Preclinical studies demonstrated that ulotaront is a full TAAR1 agonist and a 5-HT1A agonist, although it can also bind to 5-HT1B and 5-HT7 receptors, while it has no effects on D2 or 5-HT2A receptors [29]. However, it was proposed that TAAR1 agonist could inhibit D2 receptor activity through TAAR1/D2 heterodimer formation [30]. In addition, the modulation of the dopaminergic system and the ability to reduce ventral tegmental area (VTA) firing and dopaminergic release led to experimenting with TAAR1 agonists as a potential treatment in schizophrenia and psychotic disorders [31]. In a double-blind, randomized, placebo-controlled phase II clinical trial, ulotaront was administered at 50 or 75 mg/day dosages to patients with schizophrenia for 4 weeks, with a significant reduction in Positive and Negative Symptoms Scale (PANSS) scores [32]. After that, a six-month open-label extension study was carried out in patients who completed the 4 weeks of randomized double-blind trial. Ulotaront was used at doses ranging from 25 to 75 mg/day, and it caused a reduction in PANSS and in Montgomery–Åsberg Depression Rating Scale (MADRS) scores, with an improvement in depressive symptoms that are often present in patients with schizophrenia and are associated with poor functioning. From a safety standpoint, patients treated with ulotaront did not experience significant changes in body weight or other metabolic parameters, and they did not manifest extrapyramidal symptoms or prolactin level increments [33]. The pharmacokinetic (PK) profile of ulotaront was characterized in a population PK analysis in patients with schizophrenia, pooling data from phase I and II trials, in which the drug PK was described best by a two-compartment model with first-order absorption. The main meaningful covariate influencing ulotaront PK appeared to be body weight, with rapid absorption, a median Tmax of 2.8 h, a median half-life of about 7 h, and a hepatic metabolism mediated by CYP2D6 [34]. Phase III clinical trials on ulotaront efficacy and safety are ongoing, with some completed in US and EU clinical trial registries; however, the results are often still not public. A six-week phase III, double-blind, placebo-controlled, randomized clinical trial for the use of ulotaront in acute psychosis in 464 adult patients with schizophrenia reported the first results on the ability of ulotaront to reduce PANSS and Clinical Global Impressions-Severity of illness (CGI-S) scores on clinicaltrialsregister.eu, with discouraging results: only the higher dose (100 mg/day) of ulotaront was able to reduce significantly PANSS scores, and there were no significant changes in CGI-S scores (Eudra CT number: 2019-000697-37). Another phase III clinical trial on 475 adult patients with schizophrenia compared the long-term (52 weeks) safety and tolerability of ulotaront using quetiapine as an active comparator, and the preliminary results posted showed a higher number of patients experiencing adverse events related to schizophrenia in patients taking ulotaront compared to patients in the quetiapine group (NCT04115319, Eudra CT number: 2019-002259-40). Nonetheless, none of the phase III clinical trials resulted in a final publication yet, and it is possible that these disheartening results could be disconfirmed. Lastly, there are currently clinical trials studying the possibility of using ulotaront in other neuropsychiatric disorders. In particular, an ongoing phase II/III clinical trial (NCT05593029) is recruiting patients with major depressive disorder, and another one (NCT05729373) is recruiting patients with generalized anxiety disorder to determine efficacy and safety. The results from a pilot study on the use of ulotaront in patients with Parkinson’s Disease (PD) were recently published, showing promising results in patients with psychotic PD [35].

Another drug with a similar profile is ralmitaront (RO-6889450), a TAAR1 partial agonist. Compared to ulotaront, it has a more complex chemical structure, and an MW of 314.38 g/mol. In vitro research comparing ulotaront and ralmitaront efficacy showed a lower efficacy of ralmitaront on TAAR1 receptors, a lack of efficacy on 5-HT1A receptors, and a slower kinetic [36]. Two phase II clinical trials were conducted on ralmitaront (NCT03669640 and NCT04512066) to assess the efficacy in reducing negative symptoms and to evaluate its efficacy in acute psychosis in schizophrenia and schizoaffective disorder, respectively. The first trial (NCT03669640) included 131 adult patients with clinically stable schizophrenia, who had previously received no more than two different antipsychotic medications, and patients were treated with either placebo or ralmitaront for 12 weeks. The trial was prematurely terminated because it did not meet its primary endopoint, which was a reduction in the Brief Negative Symptoms Scale (BNSS) scores. The second RCT (NCT04512066), on the other hand, included 287 patients with acute exacerbation of schizophrenia or schizoaffective disorder, treated for 4, 12, or 48 weeks. The researchers compared ralmitaront (up to 150 mg/day) to placebo and risperidone (4 mg/day), failing to cause a significant reduction in PANSS scores. Other TAAR1 agonists that are under investigation are RO5203648 and RO5263397, which are currently at the preclinical stage of research, and proposed as treatment in substance-related disorders [37,38].

The good safety profiles of these compounds should encourage future research to confirm the efficacy of TAAR1 agonists for the treatment of schizophrenia or other neuropsychiatric conditions (the drugs and their most relevant clinical trials are summarized in Table 1). In fact, TAAR1 agonists are currently undergoing preclinical or clinical trials or have a potential application for other neuropsychiatric conditions, like mood disorders, anxiety disorders, substance use disorders, and post-traumatic stress disorder [39,40,41].

### 2.2. Dopaminergic and Serotoninergic Partial Agonists or Antagonists

Brilaroxazine (RP5063, MW: 450.36 g/mol) is an antipsychotic with a structure similar to aripiprazole, with the exception of the substitution of a methylene group with an oxygen atom in the quinolone ring system. Brilaroxazine has a unique pharmacological profile: it acts as a partial agonist on dopaminergic D2, D3, and D4 receptors and on serotoninergic 5-HT1A and 5-HT2A receptors. Additionally, it exhibits antagonist activity at 5-HT2B, 5-HT6, and 5-HT7 receptors [42]. The activity on D2 and D3 receptors may exert positive effects on cognition, as seen with other antipsychotics with similar mechanisms of action such as cariprazine, while the D4 partial agonism was shown to enhance memory in preclinical studies on rodents [43,44]. Brilaroxazine completed phase I and II trials and is currently undergoing phase III evaluations. In a phase II double-blind clinical trial (NCT01490086), researchers evaluated the safety, efficacy, and pharmacokinetic properties of brilaroxazine in adult patients with schizophrenia or schizoaffective disorder during an acute exacerbation of the disease. For this trial, 234 adult patients were recruited. Used at 50 mg/day, brilaroxazine was able to reduce PANSS score significantly compared to placebo, and it also improved secondary outcomes, such as CGI score and cognitive evaluations. From a safety standpoint, brilaroxazine showed a favorable profile. However, it is important to note that this trial lasted only 28 days, which may limit longer term efficacy and safety evaluations [45]. Currently, brilaroxazine is undergoing phase III trials, and in particular, the phase III clinical trial (NCT05184335) is ongoing, which is a double-blind randomized placebo-controlled clinical trial of 28 days in patients with a schizophrenia diagnosis in an acute state of the disease, followed by an open-label follow-up study of 52 weeks. The study is recruiting patients, and it is estimated to be completed by the end of 2024.

Another novel compound with antipsychotic properties is F17464 (*N*-(3-{4-[4-(8-Oxo-8H-[1,3]-dioxolo-[4,5-g]-chromen-7-yl)-butyl]-piperazin-1-yl}-phenyl)-methanesulfonamide, hydrochloride), a D3 receptor preferential antagonist and 5-HT1A receptor partial agonist with an MW of 536.04 g/mol [46]. Its activity on D3 receptors, which are homologous to D2 receptors, could explain the antipsychotic activity and may exert some positive effects on the cortical dopaminergic tone, while the partial agonist action on 5-HT1A receptors could provide pro-cognitive effects [47,48]. F17464 completed a phase II clinical trial (NCT02151656) in 158 adult patients with schizophrenia during an acute psychotic exacerbation, administered at a 40 mg/day dosage for six weeks. F17464 could significantly reduce total PANSS scores compared to placebo, with an incidence of adverse events that was just slightly higher than placebo. The most common adverse events included insomnia, agitation, and triglycerides increase, while patients did not experience any significant weight changes or extrapyramidal symptoms (EPSs) [49]. However, because antipsychotic medications are usually intended for chronic treatments, further trials should evaluate long-term safety and efficacy of this compound.

LB-102 (*N*-methylamisulpride, MW: 383.506 g/mol) is a benzamide derivative and the *N*-methylated form of amisulpride [50]. LB-102 acts as a D2/D3 and a 5-HT7 receptor antagonist. It has completed a phase I clinical trial (NCT04187560) in which 64 healthy subjects were administered different dosages of the drug to test its safety for 15 days. After the onset of two cases of EPSs at 200 mg/day, the cohort dose was decreased to 150 mg daily. Overall, LB-102 was well tolerated, although a transient increment of prolactin levels was observed in the majority of the subjects [51]. Amisulpride is known to cause hyperprolactinemia in patients, even at low dosages, and it will be important to evaluate the prevalence and the severity of hyperprolactinemia during the treatment with LB-102, which may limit its usability in the future [52]. Currently, a 28-day, phase II randomized double-blind placebo-controlled clinical trial on patients with acute schizophrenia is ongoing, intending to recruit 350 patients (NCT06179108).

Beyond these novel compounds, it is also important to mention in this category that new formulations of long-acting injectable antipsychotics (LAIs), like paliperidone palmitate 6-month (PP6M) formulation, or risperidone implants, are currently undergoing clinical trials [53,54]. Table 2 provides a summary of these novel compounds.

## 3. Serotoninergic Antagonists and Partial Agonists

Substantial evidence highlights the critical role of the serotoninergic system in the pathogenesis of schizophrenia [55,56]. Therefore, some compounds directly targeting the serotoninergic receptors were developed in the last few decades. At the moment, a few molecules that target serotoninergic receptors, with a focus on 5-HT6 receptors, are under investigation and testing (the main compounds that were studied in schizophrenia are provided in Table 3). Through these receptors, serotoninergic antagonist should be able to increase other neurotransmitters action in the prefrontal cortex (dopamine, glutamate, and acetylcholine) while reducing the GABAergic tone in this brain area. These receptors are also implicated in cognitive function, with a pro-cognitive putative role or antagonist drugs [57].

Among the 5-HT6 antagonists, the small molecule AVN-211 (CD-008-0173, Avisetron, 5,7-dimethyl-2-(methylthio)-3-(phenylsulfonyl)pyrazolo[1,5-*a*]pyrimidine, MW: 333.44 g/mol) completed a pilot study as an adjunctive treatment in patients with schizophrenia. This trial included 47 patients with schizophrenia stabilized with standard antipsychotic medications, treated with either AVN-211 or placebo, demonstrating positive results both in terms of antipsychotic action and pro-cognitive effects after four weeks. However, among the cognitive symptoms, only the domain of attention was assessed [58]. Another trial in 80 patients with stable schizophrenia with residual psychotic symptoms, however, yielded negative results in PANSS score reduction and other secondary outcomes after six weeks of adjunctive treatment compared to placebo. Interestingly, a subgroup analysis showed that this drug used as an adjunctive treatment could improve PANSS score in female patients compared to placebo [59].

Another 5-HT6 antagonist, Lu AE58054 (idalopirdine, [2-(6-fluoro-1H-indol-3-yl)-ethyl]-[3-(2,2,3,3-tetrafluoropropoxy)-benzyl]-amine, MW: 398.377 g/mol), showed promising effects in a phencyclidine model of schizophrenia in rats [60], underwent a phase II clinical trial in patient with stable schizophrenia as an adjunctive treatment to risperidone (4–8 mg/day) (NCT00810667). This trial included 164 patients, treated for 12 weeks with either Lu AE58054 or placebo, demonstrating no improvement in PANSS scores compared to placebo [61].

**Table 3:** Serotoninergic antagonists.

Drug	Mechanism of Action	Most Relevant Preclinical or Clinical Trials
AVN-211 (CD-008-0173, Avisetron)	5-HT6 antagonist	[58,59] (phase II)
Lu AE58054 (idalopirdine)	5-HT6 antagonist	NCT00810667 (phase II)
SB-742457 (intepirdine)	5-HT6 antagonist	[62] (preclinical)
WAY-181187	5-HT6 agonist	[62] (preclinical)
SYN-120 (RO5025181, landipirdine)	5-HT6 and 5-HT2 antagonist	NCT02258152 (phase II)
Masupirdine (SUVN-502)	5-HT6 antagonist	NCT02580305 (phase II) NCT05397639 (phase III)

Some compounds are undergoing preclinical evaluation, like the 5-HT6 agonist WAY-181187 (2-(1-{6-Chloroimidazo[2,1-b][1,3]thiazole-5-sulfonyl}-1*H*-indol-3-yl)ethan-1-amine, MW: 380.87 g/mol) and the 5-HT6 antagonist SB-742457 (intepirdine, 3-(benzenesulfonyl)-8-piperazin-1-ylquinoline, MW: 353.44), that were used in rats along with haloperidol or risperidone, in order to evaluate their effects on depressive and anxious symptoms, which are both impactful symptoms in patients with schizophrenia. In the study by Fernandez and colleagues, the co-administration of haloperidol and WAY-181187 yielded the best results, with a reduction in depressive-like and anxiety-like behaviors in the animals [62].

Other drugs with a similar pharmacodynamic profile are currently under investigation for the treatment of psychotic symptoms in the context of neurodegenerative disorders such as PD and Alzheimer’s Disease (AD). For example, SYN-120 (RO5025181, landipirdine, [(1*R*)-6-(3-fluorophenyl)sulfonyl-1,2,3,4-tetrahydronaphthalen-1-yl]methylurea, MW: 362.4 g/mol) is a dual 5-HT6 and 5-HT2A receptors antagonist that was tested in a 16-week phase II clinical trial (NCT02258152) involving 82 patients with PD, but unfortunately, it did not determine an improvement in the majority of studied outcomes compared to placebo, including the Scale for Assessment of Positive Symptoms adapted for PD (SAPS-PD) score, which is a questionnaire that evaluates psychotic symptoms, and the Cognitive Drug Research Computerized Cognition Battery, which evaluates continuity of attention [63]. Another selective 5-HT6 receptor antagonist is masupirdine (SUVN-502, 1-(2-bromophenyl)sulfonyl-5-methoxy-3-[(4-methylpiperazin-1-yl)methyl]indole, MW: 478.41 g/mol), which completed a phase II clinical trial in patients with agitation and psychosis in AD (NCT02580305). This trial included a vast number of patients (564), treated for 26 weeks. Masupirdine was used as an adjunctive treatment to donepezil and memantine in this double-blind, placebo-controlled clinical trial. Masupirdine was able to significantly reduce symptoms in a subgroup of patients showing neuropsychiatric manifestations like aggression/agitation and psychosis. Treatment was well tolerated, and the most common treatment emergent adverse events were urinary trait infections, falls, diarrhea, and headache [64]. A multicenter 12-week phase III clinical trial is ongoing, with expected completion in 2025, and with the intention to recruit 375 patients (NCT05397639).

Overall, this field of research remains underdeveloped, with only a few possible candidates likely to achieve approval. These drugs are most likely to be used as adjunct treatment and not as monotherapy, but they could offer some benefits in the treatment of psychotic manifestations in the context of neurodegenerative diseases.

## 4. Glutamate Modulators

Metabotropic glutamate receptors (mGluRs) are important for GABAergic and monoaminergic modulation, and they are expressed in several brain regions, like the amygdala, the hippocampus, and the prefrontal cortex. Their distribution points out to their crucial role and their association with neuropsychiatric disorders, such as schizophrenia [65,66]. For this reason, in the last decade, some drugs targeting mGluRs were developed. The main drugs in this category can be found in Table 4.

The prodrug pomaglumetad methionil (POM, LY-2140023, (1*R*,4*S*,5*S*,6*S*)-4-[[(2*S*)-2-amino-4-methylsulfanylbutanoyl]amino]-2,2-dioxo-2λ^6^-thiabicyclo[3.1.0]hexane-4,6-dicarboxylicacid;hydrate, MW: 384.4 g/mol), a methionine amide, and its active moiety LY-404039 are mGluR2/3 agonists. In the early 2010s, POM reached phase III clinical trials. However, its production was stopped after the drug failed to demonstrate its antipsychotic activity [67]. In a phase III clinical trial against aripiprazole (NCT01328093), the researchers tried to demonstrate the better tolerability of POM compared to aripiprazole. The study included 678 patients treated for 24 weeks either with POM (40 mg/day) or aripiprazole (15 mg/day). Although POM was associated with a greater weight loss after 24 weeks, it also resulted in more discontinuations due to adverse events and to a smaller reduction in PANSS scores than aripiprazole, and the trial was terminated before completion [68]. Pooled analysis of different clinical trials (NCT00149292, NCT00520923, NCT01086748, NCT01307800, and NCT01125358) showed that patients who were early-in-disease or were pre-treated with a D2 antagonist had a better response to POM, indicating that this drug could have some potential in a selected subgroup of patients [69]. In more recent years, POM was re-evaluated again in a preclinical trial in rats, that showed the ability for this drug to normalize dopaminergic neuron activity in conditions of hippocampal hyperactivity. This finding could indicate that patients who do not adequately respond to traditional antipsychotics may respond to a substance that regulates the excitation/inhibition imbalance [70].

MGS0274 besylate (TS-134, MW: 587.66 g/mol) is another mGluR2/3 agonist. It is an ester-based lipophilic prodrug that is converted to its active moiety MGS0008 and is considered a candidate for the treatment of schizophrenia [71]. In phase I single-ascending dose and multiple-ascending dose trials involving 33 and 59 healthy volunteers, respectively (NCT03746067 and NCT03742791), MGS0274 was extensively converted to MGS0008 and exhibited a low incidence of adverse events [72]. Both POM and MGS0274 were also recently evaluated in two phase I clinical trials (NCT02919774 and NCT03141658) in a ketamine-induced psychotic state in healthy volunteers. In the NCT02919774 clinical trial, 95 healthy volunteers pretreated with ketamine were administered either POM or placebo for ten days. In the NCT03141658 trial, 63 volunteers were pre-treated with ketamine, and then with MGS0274 or placebo for six days. POM led to significant effects on some clinical symptoms, while MGS0274 showed better results compared to placebo. These findings support the potential of this pharmacological class for the treatment of psychotic disorders [73].

Evenamide (NW-3509, NW-3509A, 2-[2-(3-butoxyphenyl)ethylamino]-*N*,*N*-dimethylacetamide, MW: 278.396 g/mol) exhibits quite a different mechanism of action that leads to glutamate modulation. This drug is a small molecule that can inhibit voltage-gated sodium channels, thereby regulating the excess of synaptic glutamate produced by the hypofunction of NMDAR, a type of ionotropic glutamatergic receptors. This action leads to a reduction in hyperexcitability of the cortex and the hippocampus, without affecting basal glutamate levels or other neurotransmitters [74]. In a six-week phase II placebo-controlled clinical trial in 161 patients with treatment-resistant schizophrenia, evenamide as an adjunctive treatment to a second-generation antipsychotic could significantly improve PANSS and CGI scores, with a good retention rate in the long term (up to 52 weeks) [75].

Iclepertin (BI 425809, [5-methylsulfonyl-2-[(2*R*)-1,1,1-trifluoropropan-2-yl]oxyphenyl]-[(1*R*,5*R*)-1-[5-(trifluoromethyl)-1,2-oxazol-3-yl]-3-azabicyclo[3.1.0]hexan-3-yl]methanone, MW: 512.4 g/mol) is a small molecule that acts as a potent glycine transporter 1 (GlyT1) inhibitor, which enhances glutamatergic signaling [76]. In a 12-week phase II clinical trial (NCT02832037), the efficacy and safety of iclepertin were evaluated against placebo in 509 patients with stable schizophrenia, with the intention to assess its efficacy in improving different cognitive domains (processing speed, verbal learning, working memory, reasoning/problem solving, visual learning, social cognition, and attention), evaluated with the Measurement and Treatment Research to Improve Cognition in Schizophrenia (MATRICS) Consensus Cognitive Battery (MCCB) overall composite T-score. Patients showed improvements in cognitive symptoms and overall good tolerability [77]. Three phase III clinical trials are currently further evaluating the efficacy and safety of iclepertin in improving cognitive symptoms in schizophrenia (NCT04846868, NCT04846881, NCT04860830).

Overall, this class of drugs still needs to be carefully evaluated, and further clinical trials are required to determine whether glutamate modulators can be effectively used as monotherapy or as an add-on therapy for the treatment of schizophrenia.

## 5. Muscarinic Modulators

Muscarinic receptors, particularly M1, M4, and M5 receptors, are probably involved in the pathogenesis of positive, negative, and cognitive symptoms in schizophrenia; therefore, these receptors are an appealing target to improve these symptoms [78]. Indeed, the most recent drug approved for the treatment of schizophrenia is xanomeline–trospium, which was approved in September 2024 by the Food and Drug Administration (FDA) [79].

Xanomeline–trospium (KarXT) is the first antipsychotic to reach the market with a completely different mechanism of action compared to the other antipsychotic classes. Xanomeline is a member of the tetrahydropyridines class and a muscarinic receptor agonist, with selectivity for M1 and M4 receptors [80]. Xanomeline (3-hexoxy-4-(1-methyl-3,6-dihydro-2*H*-pyridin-5-yl)-1,2,5-thiadiazole, MW: 281.42 g/mol) was studied for years as a potential candidate for the treatment of both schizophrenia and AD, but its development was stopped because of its gastrointestinal adverse effects [81]. Xanomeline is derived from the natural alkaloid arecoline, but with the ester group replaced on position 3 with a 1,2,5-thiadiazole [82]. Trospium (3-(2-Hydroxy-2,2-diphenylacetoxy)spiro[bicyclo[3.2.1]octane-8,1′-pyrrolidin]-1′-ium chloride, MW: 427.97 g/mol) is a panmuscarinic receptors antagonist, which is approved by the FDA and the European Medicines Agency (EMA) for patients with overactive bladder [83]. The combination of these two drugs led to a reduction of about 50% of gastrointestinal adverse events [84]. In a phase I clinical trial on 70 healthy volunteers (NCT02831231), trospium did not alter xanomeline hematic levels or its PK profile [84]. In a 5-week double-blind phase II clinical trial (NCT03697252), xanomeline–trospium was able to significantly reduce PANSS scores in 182 patients with acute psychosis compared to placebo [85]. Subsequent analyses confirmed these results: a pooled analysis of the aforementioned phase II clinical trial and two other 5-week phase III trials (NCT04659161 and NCT04738123) indicates xanomeline–trospium was able to reduce PANSS (both total and subscales) and CGI-S scores, with good tolerability [86]. The NCT04659161 trial included 252 patients with acute exacerbation of schizophrenia treated with either xanomeline–trospium or placebo for five weeks. The NCT04738123 trial evaluated xanomeline–trospium efficacy in 256 patients with acute psychosis and a diagnosis of schizophrenia, treated for five weeks with either the active compound or with placebo. Two open-label clinical trials (NCT04659174 and NCT04820309) are currently assessing the long-term (52 weeks) efficacy of this drug in order to evaluate its usability in maintenance treatment and to confirm its safety.

Emraclidine (CVL-231, 1-(2,4-dimethyl-5,7-dihydropyrrolo[3,4-b]pyridin-6-yl)-2-[1-[2-(trifluoromethyl)pyridin-4-yl]azetidin-3-yl]ethenone 390.410 g/mol) is a selective M4 positive allosteric modulator that has recently completed phase I clinical trials. In a phase Ib placebo-controlled clinical trial (NCT04136873), researchers assessed its efficacy and safety in 130 patients with schizophrenia, testing two dosages for 18 weeks: 20 mg twice daily and 30 mg once daily. Emraclidine was well tolerated, with the most common adverse effect being headache. Patients showed a significant reduction in PANSS scores at both dosages, with the 30 mg once-daily dosage being the best option combined with its PK profile [87]. Currently, an open-label phase II clinical trial is ongoing, intending to evaluate long-term efficacy of emraclidine (NCT05443724; Eudra CT number: 2022-001151-19).

NBI-1117568 is a selective M4 agonist currently under investigation as a potential antipsychotic for the treatment of schizophrenia [88]. Recently, a six-week phase II randomized double-blind clinical trial was completed, where 210 adult patients with acute schizophrenia have taken either NBI-1117568 at different dosages or placebo in order to evaluate it pharmacokinetics profile, efficacy, and safety, but the results are not yet public (NCT05545111).

ML-007 is a selective M1 and M4 agonist that has recently completed phase I clinical trials with a chemical structure that has not been disclosed yet. It is under investigation combined with a peripherally restricted anticholinergic drug (ML-007/PAC) for the minimization of its adverse effects [88]. MapLight Therapeutics, the pharmaceutical company developing ML-007, announced the successful completion of their second phase I trial in 2024, but the results have not been published yet.

Lastly, NS-136 is a selective M4 positive allosteric modulator explored as a potential treatment for schizophrenia [89]. A first-in-human phase I clinical trial in 76 healthy volunteers is currently ongoing, and its completion is expected in 2025 (NCT06345703).

This pharmacological class of antipsychotics has potential to enter the market, highlighted by the recent approval of xanomeline–trospium. If the results of the trials are confirmed by further research and by post-marketing evaluations, these drugs could represent a valid treatment option for patients with psychotic disorders. Table 5 summarizes the main drugs in this category.

## 6. Modulators of the Endocannabinoid System

Interestingly, cannabidiol (CBD, 2-[(1*R*,6*R*)-6-Isopropenyl-3-methylcyclohex-2-en-1-yl]-5-pentylbenzene-1,3-diol, MW: 314.469 g/mol) was proposed for the treatment of schizophrenia. CBD targets the endocannabinoids system (ECS), which is hypothesized to play a role in the pathogenesis of schizophrenia [90]. While chronic cannabis use was associated with psychotic symptom exacerbation and with an increased risk of developing schizophrenia, these effects are probably caused by Δ9 -tetrahydrocannabinol (THC). In contrast, CBD is thought to have antipsychotics effects via the enhancement of anandamide signaling [91]. The mechanism of action of CBD is complex and not completely understood. It is known that CBD interacts with various intracellular pathways, antagonizing PI3K/AKT, JAK/STAT, and MAPK/ERK; it inhibits pro-inflammatory mediators DNA transcription via the PPARγ receptor; CBD can also indirectly act on cannabidiol receptors, although it has low binding affinity for both CB1 and CB2 receptors, and it alters the uptake of GABA and adenosine, increasing their signaling [92].

Various clinical trials involving CBD in the last few years have generated mixed results in terms of positive, negative, and cognitive symptom reduction [93]. CBD has been proposed in different formulations (GWP42003; GWP42003-P) and used in clinical trials both as a monotherapy and as an add-on treatment. A 12-week phase II clinical trial comparing CBD to placebo in 95 patients with schizophrenia who were experiencing inadequate response to standard antipsychotic treatment was terminated early for lack of efficacy (NCT04421456). Another 6-week phase II placebo-controlled clinical trial on 88 patients with schizophrenia (NCT02006628) yielded more positive results. In this trial, CBD was used as an adjunctive treatment to standard antipsychotics, and patients showed a reduction in positive symptoms measured with PANSS [94]. Conversely, another phase II clinical trial NCT00588731 that assessed CBD as an add-on therapy to standard antipsychotic medications found no benefits in terms of PANSS reduction or cognitive improvement in 41 patients after six weeks of treatment compared to placebo [95]. Currently, there are few clinical trials ongoing to test the efficacy of CBD as monotherapy or as an adjunct treatment in early psychosis in youths (NCT04411225; NCT04105231; NCT02926859).

In this section, we focused specifically on CBD formulations, but other than CBD, it is important to remember that there are various allosteric modulators of the CB1 receptor undergoing clinical trials. For example, rimonabant (SR141716) is a CB1 antagonist/inverse agonist that was tested as a therapeutic option for schizophrenia, especially for cognitive symptoms, unfortunately with disappointing results [96,97]. Nonetheless, it would be important to not dismiss this class of drugs, as further clinical trials are needed to determine whether or not cannabinoid receptor modulators can be included in the treatment of schizophrenia. An overview of CBD formulations can be found in Table 6.

## 7. Mixed/Other Receptors Modulators

Some investigational antipsychotics under development target other receptors beyond the conventional ones, like the orexin or σ receptors. One of these drugs is roluperidone (MIN-101, 2-[[1-[2-(4-Fluorophenyl)-2-oxoethyl]piperidin-4-yl]methyl]-3*H*-isoindol-1-one, MW: 366.436 g/mol), a cyclic amide derivative that acts as a 5-HT2A, σ2, and α1A receptors antagonist, developed to address negative symptoms in schizophrenia [98]. In a 12-week double blind followed by a 40-week open-label extension phase III placebo-controlled clinical trial on 515 patients with significant negative symptoms of schizophrenia (NCT03397134), roluperidone was able to reduce the scores of the PANSS-derived Negative Symptom Factor Score (NSFS), although statistical significance was achieved only for the modified intent-to-treat (mITT) population. The secondary endpoint of a change in the Personal and Social Performance scale (PSP) total score was statistically significant, suggesting that roluperidone may improve negative symptoms in patients with schizophrenia [99]. A research group used network analysis to try to explain the improvements observed in negative symptoms in patients from a phase IIb and later from a phase III clinical trial. Their findings indicated that this drug was able to directly act on avolition, suggesting that this action could have effects on the other negative symptoms too [100]. Regarding roluperidone safety, a pooled analysis of two RCTs showed that the drug was well tolerated, with no significant modifications in metabolic indexes, weight, or vital signs, and it did not provoke EPSs [101].

Deudextromethorphan/quinidine (d-DXM/Q, AVP-786, CTP-786) is a combination drug of deudextromethorphan hydrobromide ((1*S*,9*S*,10*S*)-4-(trideuteriomethoxy)-17-(trideuteriomethyl)-17-azatetracyclo[7.5.3.0^1,10^.0^2,7^]heptadeca-2(7),3,5-triene;hydrate;hydrobromide, MW: 376.4 g/mol) and quinidine ((*S*)-[(2*R*,4*S*,5*R*)-5-ethenyl-1-azabicyclo[2.2.2]octan-2-yl]-(6-methoxyquinolin-4-yl)methanol, MW: 324.4 g/mol) proposed for the treatment of schizophrenia and the behavioral and psychological symptoms of AD [102]. The mechanism of action of d-DXM/Q is complex, as this compound is a σ1 receptors agonist, inhibits the reuptake of serotonin and norepinephrine (SNRI), and is an uncompetitive NMDA receptors antagonist, a muscarinic receptors agonist, and a neuronal nicotinic α3β4 receptor antagonist [103]. It was tested for the treatment of negative symptoms in schizophrenia, but a 15-week placebo-controlled phase II/III trial on 136 patients was terminated in 2023 based on futility (NCT03896945).

Zelatriazin (NBI-1065846, TAK-041, 2-(4-oxo-1,2,3-benzotriazin-3-yl)-*N*-[(1*S*)-1-[4-(trifluoromethoxy)phenyl]ethyl]acetamide, MW: 392.338 g/mol) is a GPR139 agonist. GPR139 is an orphan GPCR, especially expressed in the habenula, which is a region in the brain that is often altered in patients with schizophrenia. It was hypothesized that GPR139 agonism could improve this region’s functioning, with benefits in negative symptoms, especially in anhedonia, with promising results in animal models [104,105]. A 38-week phase II clinical trial tried to determine whether TAK-041 could change the scores of the Brief Assessment of Cognition in Schizophrenia (BACS), but there were no statistical differences compared to placebo, although the sample size of this trial included only 23 patients (NCT03319953). Takeda, the pharmaceutical company that developed TAK-041, announced the discontinuation of its development for the treatment of anhedonia in schizophrenia and major depressive disorder in 2023.

Lastly, we discussed the selective orexin-1 receptor (Ox1R) antagonist CVN766 (*N*-[(1*S*,2*S*)-2-[[3-ethyl-5-(trifluoromethyl)pyrazin-2-yl]amino]cyclopentyl]-3-(triazol-2-yl)pyridine-2-carboxamide, MW: 446.43 g/mol). Orexin A, a neuropeptide, was observed to be elevated in a subgroup of patients with schizophrenia, and it was associated with milder negative and disorganized symptoms [106]. This indicates that antagonizing Ox1R could be beneficial in patients across multiple domains of their disease. Preclinical data in rodents suggest a good permeability of CVN766 in the CNS and high selectivity for Ox1R compared to Ox2R [107]. Currently, this drug has completed successfully a phase I clinical trial on 64 healthy volunteers with the aim of evaluating its safety (NCT05105243), and the developing company Cerevance has announced a plan to move forward to phase II clinical trials.

Although not all drugs in this category have shown positive results, there are indeed promising compounds that may reach the market in the next years, like roluperidone and possibly CVN766, that confirm the need to move towards drugs with other mechanisms of action in order to treat especially negative and cognitive symptoms in schizophrenia areas that represent an unmet need in the treatment of this complex disease. Table 7 summarizes the compounds described in this section.

## 8. Phosphodiesterase (PDE) Inhibitors

Phosphodiesterases (PDEs) are enzymes involved in the catalysis of cyclic nucleotides such as cyclic AMP (cAMP) and cyclic GMP (cGMP). Their potential as targets in the treatment of schizophrenia is due to the involvement of cAMP and cGMP in the dopaminergic and glutamatergic signaling pathways [108]. Of the 11 known PDE families, PDE10A is of particular interest as it is mainly localized in the striatum, a brain region implicated in the pathogenesis of schizophrenia. Therefore, PDE10A inhibition could be exploited as a promising treatment option [109,110]. One of the first PDE10A inhibitors was PF-02545920 (MW: 392.452 g/mol), which failed a phase II clinical trial (NCT01175135) in patients with acute exacerbations of psychosis [111]. PF-02545920 was also tested in combination with a D2 antagonist in a phase Ib study, but the research was stopped due to futility [112].

TAK-063 (1-(2-fluoro-4-pyrazol-1-ylphenyl)-5-methoxy-3-(2-phenylpyrazol-3-yl)pyridazin-4-one, MW: 428.427 g/mol) is another PDE10A inhibitor that failed to meet the primary endpoint (PANSS reduction) in a randomized, placebo-controlled six-week phase II trial including 164 patients with stable schizophrenia and residual negative/cognitive symptoms (NCT02477020) but reached statistical significance in CGI score reduction [113]. Similarly, two phase II clinical trials on Lu AF11167, a selective inhibitor of PDE10A, were terminated due to the lack of superiority over placebo in the reduction in BNSS scores [114].

Recently, a novel PDE10A inhibitor, MK-8189 (2-Methyl-6-[[(1*S*,2*S*)-2-(5-methylpyridin-2-yl)cyclopropyl]methoxy]-*N*-[(5-methyl-1,3,4-thiadiazol-2-yl)methyl]pyrimidin-4-amine, MW: 382.49 g/mol), has completed phase I clinical trials. This molecule has a different structure than previous compounds, characterized by an isometric pyrimidine series that improved its PK profile, physicochemical properties, and off-target activities, and may have the potential to overcome the problems encountered with other PDE10A inhibitors [115]. MK-8189 is undergoing a phase IIb clinical trial to evaluate its efficacy and safety compared to placebo in 500 patients with schizophrenia during an acute episode (NCT04624243).

PDE9 is another PDE that might be involved in the pathogenesis of schizophrenia, with notable expression in the hippocampus and the neocortex [116]. BI 409306 (osoresnontrine, 1-(oxan-4-yl)-6-(pyridin-2-ylmethyl)-5*H*-pyrazolo[3,4-d]pyrimidin-4-one, MW: 311.345 g/mol), a highly selective PDE9 inhibitor, improved cognitive symptoms in preclinical models via increased cGMP [117]. However, this promising effect did not translate to clinical setting, as the drug failed a 20-week placebo-controlled phase II clinical trial (NCT02281773) that tried to determine its ability to improve cognitive symptoms in 518 patients with schizophrenia [118].

Despite the described setbacks in this class of drugs, the potential future application of PDE inhibitors in the treatment of schizophrenia cannot be ruled out. The synthesis of compounds with different structure, like MK-8189, may solve the problems faced with previous drugs. A summary of the PDE inhibitors described in this section can be found in Table 8.

## 9. D-Amino Acid Oxidase (DAAO) Enzyme Inhibitors

Luvadaxistat (TAK-831, 6-[2-[4-(Trifluoromethyl)phenyl]ethyl]-1,2-dihydropyridazine-3,4-dione, MW: 284.238 g/mol) is a D-amino acid oxidase (DAAO) inhibitor. DAAO inhibition increases D-serine levels, thereby enhancing NMDA receptor function, and this, in turn, could determine a possible improvement in negative symptoms as demonstrated in a rodent model [119]. In an 8-day phase II crossover clinical trial including 31 patients with stable schizophrenia (NCT03359785), researchers found an improvement in mismatch negativity in patients treated with luvadaxistat compared to placebo, which may predict an improvement in cognitive dysfunction. Unfortunately, researchers did not find an improvement in their primary endpoint measure (eyeblink conditioning, which is a measure of learning); however, it is to be noted that the sample size of this study was small and that treatment duration was only 8 days [120]. Another phase II clinical trial in 216 patients with cognitive impairment associated with schizophrenia is ongoing, with completion expected in 2025 (NCT05182476).

Sodium benzoate (the sodium salt of benzoic acid, MW: 144.105 g/mol), another DAAO inhibitor, has already shown promising results in improving cognitive symptoms in AD [121]. Interestingly, a recent systematic review and meta-analysis showed that sodium benzoate may be effective for the improvement in positive symptoms rather than negative or cognitive ones in patients with schizophrenia. However, this finding was based on only four studies, and the authors emphasized the need for future research. In particular, the authors found only 26 studies that could potentially be screened for the systematic review, which underlines the scarcity of the literature on sodium benzoate in the context of schizophrenia treatment, and the four included RCTs were characterized by high heterogeneity, which may limit the results’ robustness [122]. Currently, a few phase II clinical trials are evaluating the efficacy of sodium benzoate as an add-on therapy in schizophrenia (NCT03510741, NCT06340789, ACTRN12621000327886).

This quite novel category of drugs in the field of schizophrenia treatment is relatively unexplored yet, and further research is required to assess its efficacy and safety in the long term. These compounds are summarized in Table 9.

## 10. Conclusions

This review tried to summarize the actual state of the art of the latest antipsychotics developed, with a specific focus on their novel mechanism of action and current clinical status and approval from regulatory agencies. Surprisingly, there are hundreds of clinical trials studying the efficacy and safety of new drugs with putative antipsychotic properties. For instance, the association of mixed muscarinic agonist/antagonist xanomeline–trospium has just been approved for the treatment of schizophrenia, and other drugs are in advanced stages of trials and may be approved in the next few years. Unfortunately, the promising TAAR1 agonists, especially ulotaront, did not meet the expected efficacy endpoints in phase III trials, and are currently being investigated for the treatment of other neuropsychiatric conditions. On the other hand, compounds such as brilaroxazine or iclepertin, if the positive results obtained so far are confirmed in phase III clinical trials, may be approved for the treatment of schizophrenia in the next few years. Other pharmacological classes, such as PDE and DAAO inhibitors, are still at earlier phases of development, and their efficacy and safety still need to carefully be evaluated. Overall, it is important to expand the inventory of drugs available for the treatment of schizophrenia and other psychotic disorders and to focus on receptors that go beyond the dopaminergic and serotoninergic system in order to address the persisting negative and cognitive symptoms and to tailor the treatments to the patient’s specific needs. In addition, some of the new candidates seem to have a better profile in terms of safety and tolerability, and this is relevant considering that antipsychotic medications are often endowed with severe adverse effects. Another challenge in the pharmaceutical research and development of new antipsychotic drugs is their delivery and long-acting formulation in the direction of a patient-tailored therapy, which is strongly needed today.

## 11. Limitations

This review has some limitations. First, for some of the drugs included in the discussion, it was difficult to find information because the compounds are still at earlier stages of development, and there are few publications or preliminary press releases by the pharmaceutical companies. Moreover, for some clinical trials, the results are not yet public; therefore, it was not possible to retrieve more information. Another limitation concerns the study designs of some of the studies. In most cases, the treatment duration was limited to a few weeks, which may be useful to understand short-term treatment efficacy (i.e., in acute settings), but it lacks information of the long-term effects (either therapeutic or adverse effects). However, numerous compounds that have reached phase III are undergoing longer follow-up studies (up to 52 weeks) to better evaluate the efficacy in maintenance treatment and the adverse effects that may not be visible in shorter studies. Another limitation characterizing some of the phase II clinical trials is the sample size, which may limit the generalizability of the findings. Lastly, in phase II and in many available phase III clinical trials, the drugs were tested against placebo, and only in a few cases, they were tested in comparison to an active compound (usually quetiapine or aripiprazole). Some of these limitations are inevitable in earlier phases of development; therefore, we expect that in the next few years researchers will be able to overcome those issues and to consolidate some of these preliminary results.

## Figures and Tables

**Table 1:** TAAR1 agonists.

Drug	Mechanism of Action	Most Relevant Clinical Trials
Ulotaront (SEP-363856)	TAAR1 agonist 5-HT1A agonist	Eudra CT number: 2019-000697-37 (phase III) NCT04115319, Eudra CT number: 2019-002259-40 (phase III)
Ralmitaront (RO-6889450)	TAAR1 partial agonist	NCT03669640 (phase II) NCT04512066 (phase II)

**Table 2:** Novel atypical antipsychotics.

Drug	Mechanism of Action	Most Relevant Clinical Trials
Brilaroxazine (RP5063)	D2, D3, and D4 partial agonist 5-HT1A and 5-HT2A partial agonist 5-HT2B, 5-HT6, and 5-HT7 antagonist	NCT01490086 (phase II) NCT05184335 (phase III)
F17464	D3 antagonist 5-HT1A partial agonist	NCT02151656 (phase II)
*N*-methylamisulpride (LB-102)	D2 and D2 antagonist 5-HT7 antagonist	NCT04187560 (phase I) NCT06179108 (phase II)

**Table 4:** Glutamatergic modulators.

Drug	Mechanism of Action	Most Relevant Clinical Trials
Pomaglumetad methionyl (POM, LY-2140023) LY-404039 (active moiety)	mGluR2/3 agonists	NCT01328093 (phase III) NCT02919774 (phase I)
MGS0274 besylate (TS-134) MGS0008 (active moiety)	mGluR2/3 agonists	NCT03746067 (phase I) NCT03742791 (phase I) NCT03141658 (phase I)
Evenamide	Inhibition of voltage-gated sodium channels NMDAR regulation	NW-3509/014/II/2019 (phase II)
Iclepertin (BI 425809)	GlyT1 inhibitor	NCT02832037 (phase II) NCT04846868 (phase III) NCT04846881 (phase III) NCT04860830 (phase III)

**Table 5:** Muscarinic modulators.

Drug	Mechanism of Action	Most Relevant Clinical Trials
Xanomeline–trospium (KarXT) (Approved in September 2024)	M1 and M4 agonist (xanomeline), pan-mAChR antagonist (trospium)	NCT03697252 (phase II) NCT04659161 (phase III) NCT04738123 (phase III)
Emraclidine (CVL-231)	M4 positive allosteric modulator	NCT04136873 (phase Ib) NCT05443724; Eudra CT number: 2022-001151-16 (phase II)
NBI-1117568	M4 agonist	NCT05545111 (phase II)
ML-007	M1 and M4 agonist	NA
NS-136	M4 positive allosteric modulator	NCT06345703 (phase I)

**Table 6:** Endocannabinoid system modulators.

Drug	Mechanism of Action	Most Relevant Clinical Trials
CBD GWP42003 GWP42003-P	Increased anandamide signaling Increased GABA and adenosine tone PI3K/AKT, JAK/STAT, and MAPK/ERK pathways antagonism Inhibition of pro-inflammatory mediators	NCT04421456 (phase II) NCT02006628 (phase II) NCT00588731 (phase II) NCT04411225 (phase III) NCT04105231 (phase II) NCT02926859 (phase II)
Rimonabant (SR141716)	CB1 antagonist/inverse agonist	NCT00547118 (phase II)

**Table 7:** Putative antipsychotics that target other receptors.

Drug	Mechanism of Action	Most Relevant Clinical Trials
Roluperidone (MIN-101)	5-HT2A antagonist σ2 antagonist α1A antagonist	NCT03397134 (phase III)
Deudextromethorphan/quinidin	σ1 agonist SNRI NMDA uncompetitive antagonist mAChR agonist nicotinic α3β4 antagonist	NCT03896945 (phase II/III) [terminated]
TAK-041	GPR139 agonist	NCT03319953 (phase II)
CVN766	Ox1R antagonist	[108] (preclinical) NCT05105243 (phase I)

**Table 8:** PDE inhibitors.

Drug	Mechanism of Action	Most Relevant Clinical Trials
PF-02545920	PDE10A inhibitor	NCT01175135 (phase II) NCT01939548 (phase Ib) NCT01829048 (phase II)
TAK-063	PDE10A inhibitor	NCT02477020 (phase II)
Lu AF11167	PDE10A inhibitor	NCT03793712 (phase II) [terminated] NCT03929497 (phase II) [terminated]
MK-8189	PDE10A inhibitor	NCT05406440 (phase I) NCT04624243 (phase IIb)
BI-409306 (osoresnontrine)	PDE9 inhibitor	NCT02281773 (phase II)

**Table 9:** Enzyme inhibitors.

Drug	Mechanism of Action	Most Relevant Clinical Trials
Luvadaxistat (TAK-831)	DAAO inhibitor	NCT03359785 (phase II) NCT05182476 (phase II)
Sodium benzoate	DAAO inhibitor	ACTRN12621000327886 (phase II) NCT03510741 (phase II) NCT06340789 (phase II)
